# Radiotherapy and immune microenvironment crosstalk in pancreatic cancer: a comprehensive review of current insights and future directions

**DOI:** 10.3389/fimmu.2025.1619946

**Published:** 2025-07-03

**Authors:** Yucheng Xu, Jingjing Chen, Yudong Qiu, Juan Du

**Affiliations:** ^1^ Department of Pancreatic and Metabolic Surgery, Nanjing Drum Tower Hospital, Affiliated Hospital of Medical School, Nanjing University, Nanjing, Jiangsu, China; ^2^ The Comprehensive Cancer Center, Nanjing Drum Tower Hospital, Medical School of Nanjing University, Nanjing, China; ^3^ Nanjing Drum Tower Hospital Clinical College of Nanjing University of Chinese Medicine, Nanjing, China

**Keywords:** pancreatic ductal adenocarcinoma (PDAC), tumor microenvironment (TME), radiotherapy (RT), immunotherapy, immune modulation

## Abstract

Pancreatic ductal adenocarcinoma (PDAC) remains one of the most lethal malignancies, largely due to its profoundly immunosuppressive tumor microenvironment (TME) and intrinsic resistance to conventional therapies. Radiotherapy (RT), traditionally valued for its cytotoxic effects, has recently been recognized for its immunomodulatory potential. This mini-review explores the multifaceted interactions between RT and the PDAC immune microenvironment, highlighting mechanisms such as induction of immunogenic cell death, enhancement of antigen presentation, modulation of cytokine and chemokine profiles, and upregulation of immune checkpoint molecules. These effects may transform immunologically “cold” tumors into “hot” ones, providing a rationale for combination strategies with immunotherapy. However, the dense desmoplastic stroma, abundance of regulatory T cells, and myeloid-derived suppressor cells within PDAC present substantial challenges that hinder effective immune activation. Advances in single-cell and spatial transcriptomic technologies offer new opportunities to better characterize the TME and guide personalized treatment strategies. By synthesizing mechanistic insights and clinical evidence, this review underscores the potential of integrating RT with immunotherapy to overcome resistance mechanisms and improve therapeutic outcomes in PDAC.

## Introduction

Pancreatic ductal adenocarcinoma (PDAC) is a highly lethal malignancy, representing the most common form of pancreatic cancer. In the United States, it ranks as the tenth most commonly diagnosed cancer but stands as the third leading cause of cancer-related deaths (1). This disparity underscores the aggressive nature of PDAC and the challenges associated with its management.

Over the past decade, there has been a modest improvement in the overall survival (OS) rates for PDAC patients. The five-year relative survival rate has increased from 7% in 2015 to approximately 12% in 2023 (2). Despite this progress, the prognosis remains grim, with the majority of patients succumbing to the disease within a few years of diagnosis.

PDAC exhibits an intrinsic resistance to conventional therapies, including chemotherapy and radiation. This resistance is largely attributed to the uniquely immunosuppressive tumor microenvironment (TME) characteristic of PDAC. The TME comprises a dense desmoplastic stroma and a complex network of immunosuppressive cells and signaling molecules that collectively create a formidable barrier against effective treatment. Key components of the TME include cancer-associated fibroblasts (CAFs), myeloid-derived suppressor cells (MDSCs), tumor-associated macrophages (TAMs), and regulatory T cells (Tregs). These elements work in concert to promote tumor progression, inhibit immune surveillance, and reduce the efficacy of therapeutic agents (3).

Recent research has illuminated the intricate interplay between radiotherapy (RT) and the immune components of the TME, suggesting potential avenues to enhance treatment outcomes (4–6). Traditionally, RT has been valued for its direct cytotoxic effects on tumor cells through DNA damage. However, emerging evidence indicates that RT also exerts significant immunomodulatory effects within the TME. These effects include the induction of immunogenic cell death, enhancement of antigen presentation, modulation of immune checkpoint molecule expression, and alteration of the cytokine milieu. Collectively, these changes can potentially convert an immunologically “cold” tumor into a “hot” one, thereby enhancing anti-tumor immunity and improving therapeutic efficacy.

Despite these promising insights, the clinical application of combining RT with immunotherapy in PDAC has faced significant challenges. The intrinsic resistance mechanisms of the TME, the heterogeneity of the immune response among patients, and the potential for increased toxicity have limited the success of this combined approach. Addressing these challenges requires a deeper understanding of the TME’s complexities and the development of personalized treatment strategies that can effectively harness the synergistic potential of RT and immunotherapy.

This mini review provides a comprehensive and updated perspective on the crosstalk between RT and the immune microenvironment in PDAC. It examines therapeutic strategies that leverage the immunomodulatory effects of RT, evaluates key challenges such as tumor heterogeneity and immunosuppressive stromal barriers, and outlines future directions to improve patient outcomes. Notably, this review highlights two emerging areas that distinguish it from prior literature: the potential of RT to induce neoantigen formation, and the application of single-cell and spatial transcriptomic technologies to characterize the tumor microenvironment. By integrating recent mechanistic discoveries with advanced profiling tools, this review identifies new opportunities for designing rational combination therapies and guiding personalized treatment strategies in PDAC.

## Current landscape of radiotherapy and immunotherapy in pancreatic cancer

For patients with borderline resectable or locally advanced PDAC, radiotherapy—particularly stereotactic body radiotherapy (SBRT) and intensity-modulated radiotherapy (IMRT)—is a key component of combination therapy, frequently administered alongside systemic chemotherapy to enhance local control and increase resectability. Neoadjuvant approaches incorporating radiotherapy have been shown to improve R0 resection rates while reducing vascular and lymphatic invasion (7, 8).

Beyond its direct cytotoxic effects, radiotherapy exerts potent immunomodulatory influences. It induces immunogenic cell death, enhances tumor antigen presentation, alters the cytokine milieu, and upregulates immune checkpoint molecules, collectively shifting the tumor immune microenvironment from “cold” to “hot.” These effects provide a mechanistic rationale for combining radiotherapy with immunotherapy, particularly immune checkpoint inhibitors (ICIs), to enhance antitumor immunity in PDAC.

The therapeutic potential of radiotherapy within conversion treatment paradigms has been evaluated in key clinical trials. In the LAP07 study, patients with locally advanced PDAC were randomized to receive either chemoradiotherapy or chemotherapy alone. While no difference in overall survival was observed, the chemoradiotherapy group achieved significantly improved local tumor control (46% vs. 32%, P = 0.03), reinforcing radiotherapy’s role in stabilizing local disease and preventing progression (9–11).

Building upon these findings, the PREOPANC-2 study (12), directly compared neoadjuvant gemcitabine-based chemoradiotherapy with intensified neoadjuvant chemotherapy using FOLFIRINOX (FFX) in patients with resectable or borderline resectable PDAC. The study demonstrated that overall survival was comparable between the two regimens, with median OS reaching 21.9 months in the FFX group and 21.3 months in the GEM-based chemoradiotherapy group, with no statistically significant difference. Secondary endpoints, including R0 resection rates and disease-free survival, were likewise similar across arms. These results underscore that local radiotherapy combined with single-agent gemcitabine chemotherapy confers equivalent therapeutic efficacy to that of intensified four-drug FOLFIRINOX chemotherapy in this patient population.

Further evidence from the ESPAC-5 trial—a randomized phase II study in patients with borderline resectable PDAC—demonstrated the advantage of neoadjuvant strategies over immediate surgery (13). Patients were randomized to one of four arms: immediate surgery, gemcitabine plus capecitabine, FOLFIRINOX, or capecitabine-based chemoradiotherapy. Only 14% (3/21) of patients in the immediate surgery group achieved R0 resection, compared to 23% (7/30) across the neoadjuvant arms. Notably, R0 resection was highest (37%) in the chemoradiotherapy group. Lymph node positivity was also lowest in this group (25%) compared to 64–90% in others. One-year disease-free survival significantly favored neoadjuvant therapy (59%, 95% CI: 46–74%) over immediate surgery (33%, 95% CI: 19–58%; HR 0.53, P = 0.016), as did 1-year overall survival (76% vs. 39%; HR 0.29, P = 0.0052). While the 12-month overall survival for chemoradiotherapy (60%) was numerically lower than FOLFIRINOX (84%) and gemcitabine–capecitabine (78%), its higher R0 resection rate and lower nodal involvement suggest superior local disease control.

Taken together, these findings underscore the utility of radiotherapy—not only in enhancing resectability and local control—but also as a potential immunologic primer in PDAC. Despite encouraging mechanistic evidence, the clinical translation of radiotherapy–immunotherapy combinations remains limited, primarily due to the profoundly immunosuppressive tumor microenvironment and interpatient heterogeneity. Moving forward, multidisciplinary treatment design must incorporate mechanistic insights into radiotherapy-induced immune modulation. The integration of single-cell and spatial transcriptomic profiling may help refine patient selection and guide the development of rational, biomarker-driven radiotherapy–immunotherapy strategies for clinical application.

## The immunosuppressive tumor microenvironment in PDAC

PDAC is an exceptionally aggressive malignancy with a notably poor prognosis, largely due to its complex and immunosuppressive TME. A hallmark of PDAC’s TME is pronounced desmoplasia, characterized by the excessive accumulation of fibrous or connective tissue. This desmoplastic stroma not only forms a physical barrier that hampers drug delivery and immune cell infiltration but also actively participates in tumor progression through complex biochemical interactions. Within this dense stroma, several key cellular components contribute to the immunosuppressive landscape:

1. Cancer-Associated Fibroblasts (CAFs): These cells are pivotal in remodeling the extracellular matrix (ECM), leading to increased tissue stiffness and the formation of a physical barrier that impedes immune cell infiltration and drug delivery (14). CAFs secrete various cytokines and growth factors that promote tumor growth and further immunosuppression (15, 16).
2. Pancreatic stellate cells (PSCs): PSCs support tumor progression by secreting ECM components and pro-inflammatory cytokines, and by increasing the number of immunosuppressive cells, further hindering the infiltration of cytotoxic T cells (17).
3. Myeloid-Derived Suppressor Cells (MDSCs): MDSCs accumulate in the PDAC TME and inhibit the activation and proliferation of T cells, thereby dampening the anti-tumor immune response. Their presence is associated with poor prognosis and resistance to therapies.
4. Tumor-Associated Macrophages (TAMs): Predominantly polarized towards the M2 phenotype in PDAC, TAMs support tumor progression by promoting angiogenesis, suppressing adaptive immunity, and facilitating tissue remodeling. They release immunosuppressive cytokines such as interleukin-10 (IL-10) and transforming growth factor-beta (TGF-β) (14).
5. Regulatory T Cells (Tregs): Tregs are enriched in the PDAC TME and contribute to immune tolerance by inhibiting effector T cell functions. Their abundance correlates with reduced patient survival and diminished response to immunotherapies.

The ECM itself is heavily modified in PDAC, with increased deposition of collagen and other matrix proteins that not only create a physical barrier but also actively participate in signaling pathways that promote immunosuppression and tumor progression.

## Radiotherapy-induced modulation of the immune microenvironment

RT has long been recognized for its direct cytotoxic effects on tumor cells through the induction of DNA damage. However, emerging evidence underscores RT’s significant immunomodulatory capabilities within the TME, suggesting its potential to transform immunologically “cold” tumors—those that are poorly infiltrated by immune cells—into “hot” tumors, characterized by robust immune cell infiltration and activity. This transformation can enhance anti-tumor immunity through several mechanisms:

1. Induction of Immunogenic Cell Death (ICD)

**Figure 1:**
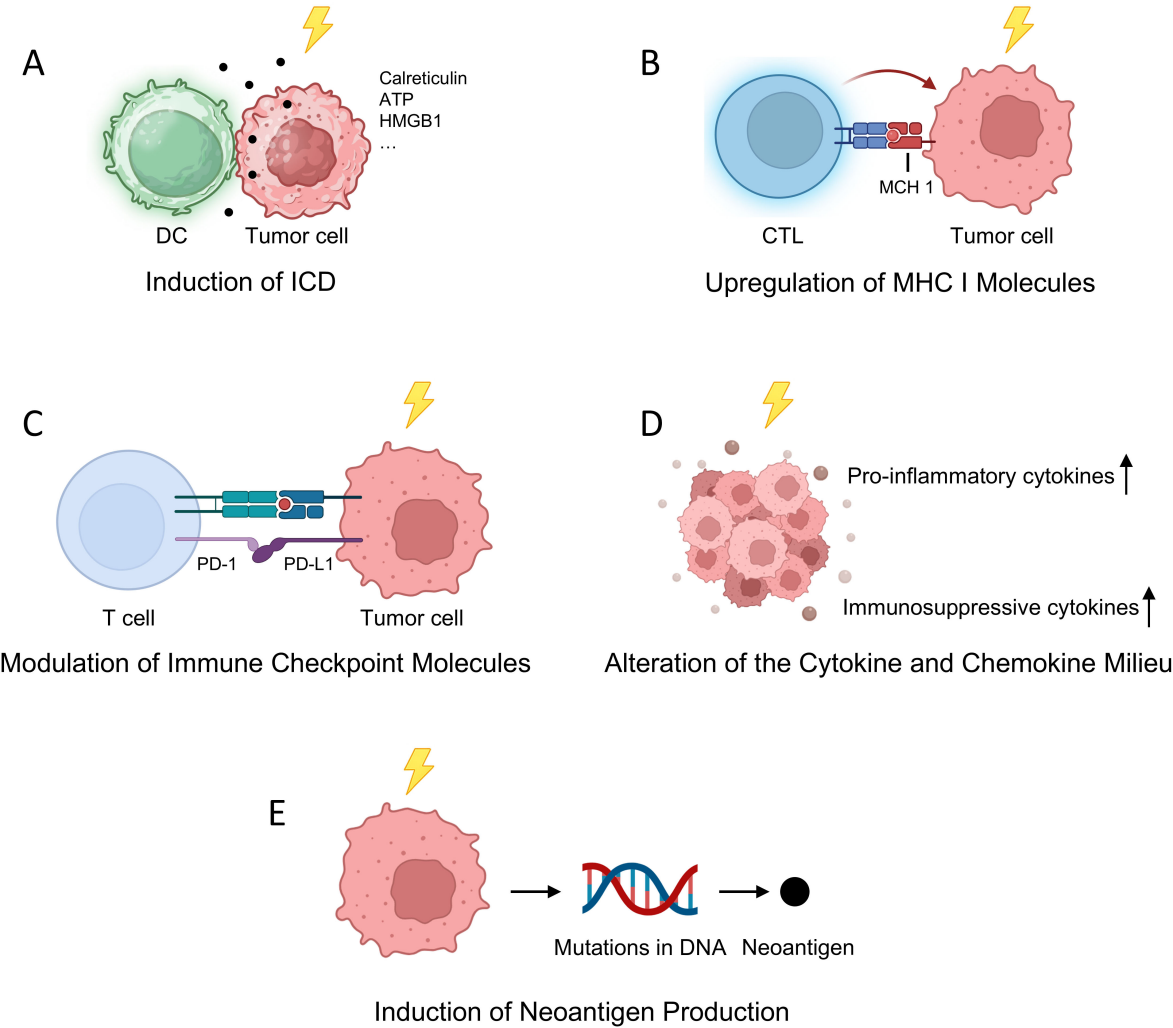
Radiotherapy-Induced Modulation of the Immune Microenvironment. **(A)** RT triggers ICD, leading to the release of DAMPs such as ATP, calreticulin, and HMGB1, which activate DCs and initiate tumor-specific adaptive immune responses; **(B)** RT upregulates MHC I molecules on tumor cells, thereby enhancing CTL-mediated tumor recognition and killing; **(C)** RT modulates the expression of immune checkpoint proteins, including PD-L1, offering a rationale for combinatorial strategies with ICIs; **(D)** RT reshapes the cytokine and chemokine milieu by promoting both pro-inflammatory and immunosuppressive factors, influencing the recruitment and polarization of immune subsets; **(E)** RT induces the formation of neoantigens by generating novel tumor-specific mutations, broadening the antigenic landscape and potentiating T cell-mediated immune surveillance.

RT can initiate ICD, a form of cell death that activates the immune system against tumor cells. During ICD, dying tumor cells release tumor-associated antigens along with danger-associated molecular patterns (DAMPs) such as calreticulin, ATP, and high-mobility group box 1 (HMGB1). These molecules serve as signals that recruit and activate dendritic cells (DCs), which process the antigens and present them to naïve T cells, thereby priming tumor-specific adaptive immune responses. This process effectively turns the tumor into an *in situ* vaccine, promoting systemic anti-tumor immunity (18) (**Figure 1A**).

2. Upregulation of Major Histocompatibility Complex (MHC) Class I Molecules

RT enhances the expression of Major Histocompatibility Complex (MHC) class I molecules on tumor cells, crucial for presenting endogenous tumor antigens to cytotoxic T lymphocytes (CTLs). This upregulation improves CTL recognition and targeting of tumor cells, facilitating more effective immune-mediated tumor destruction. Mechanistically, RT increases intracellular peptide generation and modifies the peptide repertoire, leading to enhanced MHC class I expression and the presentation of radiation-induced tumor-specific antigens. Additionally, RT activates signaling pathways such as mTOR, boosting protein synthesis and peptide availability for MHC class I loading. These processes collectively enhance the immune system’s ability to detect and eliminate tumor cells, underscoring the potential of combining RT with immunotherapies to improve anti-tumor responses (19–22) (**Figure 1B**).

3. Modulation of Immune Checkpoint Molecules

RT can influence the expression of immune checkpoint proteins, notably programmed death-ligand 1 (PD-L1), on tumor and immune cells within the TME. While upregulation of PD-L1 may contribute to immune evasion by engaging the PD-1 receptor on T cells and inhibiting their activity, this alteration also provides a therapeutic opportunity. Combining RT with ICIs that block the PD-1/PD-L1 axis can overcome this adaptive resistance mechanism, restoring T cell function and enhancing anti-tumor responses (23–25) (**Figure 1C**).

4. Alteration of the Cytokine and Chemokine Milieu

RT affects the secretion profiles of various cytokines and chemokines within the TME, which in turn influences the recruitment, activation, and polarization of immune cells. For instance, RT can increase the production of pro-inflammatory cytokines such as interferon-gamma (IFN-γ) and tumor necrosis factor-alpha (TNF-α), promoting the infiltration and activation of effector T cells and DCs. Conversely, RT may also elevate levels of immunosuppressive cytokines like transforming growth factor-beta (TGF-β), which can recruit regulatory T cells (Tregs) and myeloid-derived suppressor cells (MDSCs), potentially dampening immune responses. The net effect of RT on the cytokine milieu is complex and context-dependent, influenced by factors such as radiation dose, fractionation schedule, and the specific tumor type (26) (**Figure 1D**).

5. Induction of Neoantigen Production

RT has been shown to induce mutations in tumor cell DNA, leading to the formation of novel peptides, or neoantigens, that were not previously present in the tumor. These radiation-induced neoantigens can be recognized as foreign by the immune system, eliciting robust T cell responses. This phenomenon is particularly significant in tumors with initially low mutational burdens, as RT can broaden the repertoire of tumor antigens available for immune recognition, thereby enhancing the effectiveness of subsequent immunotherapies (21, 26, 27).

For instance, a study published in Proceedings of the National Academy of Sciences demonstrated that noncurative irradiation induces mutations in tumor cells lacking neoantigens, leading to the generation of new neoantigens that elicit protective antitumor immunity (28). Additionally, research featured in The Journal of Clinical Investigation provided evidence that radiotherapy can expose existing neoantigens to the immune system, enhancing the efficacy of neoantigen vaccination in a poorly immunogenic model of breast cancer (29). Moreover, a review in Cancer Biology & Medicine discussed how radiation therapy can enhance cancer cell antigenicity by upregulating the expression of genes involved in the response to DNA damage and cellular stress, potentially exposing immunogenic mutations to the immune system (26) (**Figure 1E**).

Importantly, in murine models, RT has been shown to upregulate the expression of genes such as CAND1, DHX58, ADGRF5, and RAET1E, leading to the formation of neoantigens that elicit specific T cell responses (29) (**Table 1**).

**Table 1:** Characteristics of RT-induced neoantigens, including their gene source, mutation type, MHC binding classification, and immune response type.

Gene	Mutation Sequence	MHC Binding Type	Immune Response Type
CAND1	Mutated Peptide	H2-K^d^	CD8^+^ T Cell Response
DHX58	Mutated Peptide	H2-L^d^	CD8^+^ T Cell Response
ADGRF5	Mutated Peptide	H2-I^d^	CD4^+^ T Cell Response
RAET1E	Mutated Peptide	H2-L^d^	CD8^+^ T Cell Response

These findings suggest that RT-induced neoantigen formation can play a crucial role in converting immunologically “cold” tumors into “hot” ones, thereby improving responses to immunotherapies.

RT has demonstrated significant immunomodulatory effects within the TME of PDAC, positioning it as a potent adjunct to immunotherapy. By altering the TME to favor immune activation and inducing the production of neoantigens, RT enhances the efficacy of immunotherapeutic agents. This synergistic interaction offers a promising avenue for improving treatment outcomes in PDAC, a cancer traditionally resistant to both RT and immunotherapy. Ongoing research aims to optimize RT protocols and combination strategies to fully exploit these interactions, with the goal of achieving more durable and effective anti-tumor responses (30).

Despite encouraging preclinical data supporting RT-immunotherapy combinations, several clinical trials have yielded disappointing results. For example, the PREOPANC-2 trial did not show a significant overall survival benefit for patients receiving gemcitabine-based chemoradiotherapy compared to FOLFIRINOX, despite mechanistic rationale for RT-mediated immune activation. These discrepancies may arise from various factors including limited immune priming in poorly immunogenic tumors, suboptimal treatment sequencing, and inadequate patient stratification. Moreover, immune-related adverse events (irAEs) and variability in tumor mutational burden may further confound clinical outcomes. A more nuanced understanding of these clinical limitations is necessary to guide future trial designs.

### Challenges in Combining Radiotherapy with Immunotherapy in PDAC

The immunosuppressive stromal and cellular components of the PDAC microenvironment, including CAFs, MDSCs, and Tregs, significantly restrict immune infiltration and activation, posing major obstacles to the success of RT-immunotherapy combinations (31, 32).

Another important consideration is the inherent limitation of current preclinical models used to study PDAC. While murine models have contributed significantly to our understanding of tumor immunology, they often fail to recapitulate key features of the human PDAC microenvironment, particularly the extensive desmoplastic stroma and complex immunosuppressive networks. These models may also lack the genetic and spatial heterogeneity seen in human tumors, limiting their translational relevance. Furthermore, *in vitro* systems rarely reflect the dynamic immune-stromal interactions that occur *in vivo*. Recognizing these gaps is essential for interpreting preclinical findings with caution and underscores the urgent need to develop more physiologically relevant models.

## Future directions

PDAC is an aggressive malignancy characterized by a unique TME that poses significant therapeutic challenges. RT is a primary treatment modality for PDAC, known for its direct induction of DNA damage in tumor cells. Beyond this, RT has demonstrated potential immunomodulatory effects that could be leveraged to enhance treatment outcomes. However, the clinical efficacy of combining RT with immunotherapy in PDAC remains suboptimal, primarily due to the immunosuppressive nature of the TME and tumor heterogeneity.

1. Optimizing Radiotherapy and Immunotherapy Combination Strategies

Future research should focus on determining the optimal sequencing and combination strategies of RT and immunotherapy to maximize synergistic effects and therapeutic efficacy. This includes investigating whether RT should be administered prior to, following, or concurrently with immunotherapeutic agents to enhance tumor antigen presentation and potentiate immune responses.

2. Development of Predictive Biomarkers

PDAC is characterized by a highly heterogeneous TME, complicating the prediction of individual responses to combined RT and immunotherapy. Consequently, there is an urgent need to identify novel biomarkers to enhance patient stratification and optimize treatment strategies. Recent studies utilizing single-cell RNA sequencing have identified tumor necrosis factor receptor superfamily member 4 (TNFRSF4), also known as OX40, as a signature gene associated with Tregs and exhausted T cell populations within the PDAC TME. Notably, elevated expression of TNFRSF4 has been correlated with poorer survival outcomes, underscoring its potential as a prognostic biomarker for guiding patient stratification and informing therapeutic decision-making (33, 34).

Despite growing mechanistic insight, significant barriers remain in translating RT-immunotherapy combinations into clinical success for PDAC. One major challenge is insufficient patient stratification, which limits the ability to identify subgroups most likely to benefit from specific treatment modalities. The lack of validated predictive biomarkers further impedes personalized treatment planning and trial enrollment efficiency. Moreover, irAEs, particularly when RT is combined with ICIs, raise safety concerns that require careful toxicity management strategies. These clinical limitations have contributed to inconsistent outcomes in trials and highlight the need for refined trial design and better biological guidance.

To address these gaps, advanced profiling technologies such as single-cell RNA sequencing (scRNA-seq) and spatial transcriptomics offer powerful tools to dissect the cellular and spatial complexity of the PDAC tumor microenvironment. These platforms can reveal rare immunoregulatory subpopulations, spatially restricted immune exclusion zones, and context-specific signaling cues, all of which are critical for designing rational combination regimens. Integrating such technologies into clinical trial frameworks can improve patient stratification, enable biomarker-driven interventions, and mitigate off-target immune toxicity by informing mechanism-based treatment schedules.

3. Remodeling the Tumor Microenvironment to Support Immune Activation

The TME in PDAC is characterized by a dense desmoplastic stroma and an abundance of immunosuppressive cell populations, including CAFs, Tregs, and MDSCs. These components collectively create a barrier that hinders immune cell infiltration and function, thereby limiting the efficacy of RT and immunotherapy. Developing strategies to remodel the TME to support effective anti-tumor immunity is imperative.

4. Integrating Advanced Technologies to Elucidate TME Dynamics

The integration of advanced technologies, such as single-cell RNA sequencing (scRNA-seq) and spatial transcriptomics, can provide deeper insights into the dynamic interactions within the TME. These technologies enable high-resolution mapping of cellular compositions and their spatial organization, offering a comprehensive understanding of the TME’s complexity. For instance, scRNA-seq allows for the characterization of individual cell populations within tumors, revealing heterogeneity and identifying rare cell types that may contribute to therapy resistance. Spatial transcriptomics adds another layer by preserving the spatial context of gene expression, elucidating how cellular interactions and localizations influence tumor progression and response to therapy (33, 35, 36).

Recent studies have demonstrated the utility of advanced profiling technologies in PDAC. Integrative analyses combining single-cell and spatial transcriptomics have revealed distinct transcriptional landscapes and cellular architectures between tumor cores and invasive margins, highlighting the impact of spatial organization on therapeutic response.

Several ongoing clinical trials are evaluating RT-immunotherapy combinations in PDAC. For instance, NCT02866383 (37) is investigating SBRT in combination with nivolumab and ipilimumab to enhance immune responses in PDAC patients. NCT04612530 (PANFIRE-III trial) (38) is assessing the safety and efficacy of irreversible electroporation (IRE) combined with nivolumab and a CpG oligonucleotide in metastatic PDAC, aiming to boost antigen release and immune activation. These studies reflect efforts to translate immunomodulatory mechanisms of RT into rational, immune-based therapeutic strategies in the clinical setting.

In parallel, managing immune-related toxicities is essential to ensure the safety of combined treatment approaches. Current strategies include early intervention with corticosteroids for high-grade irAEs, predefined dose modification protocols, and the integration of biomarker-based predictive tools to identify high-risk patients. Incorporating these measures into both clinical trials and routine practice is critical to achieving a balance between therapeutic efficacy and tolerability.

Together, these emerging technologies, clinical trials, and toxicity management strategies represent a comprehensive framework for advancing RT–immunotherapy integration in PDAC. Sustained efforts to align biological discovery with patient-centered trial design will be essential for translating innovation into durable clinical benefit.

## Conclusion

PDAC remains one of the most treatment-resistant malignancies, largely due to its immunosuppressive TME and dense desmoplastic stroma. While RT has traditionally served as a local cytotoxic modality, emerging evidence highlights its immunomodulatory effects, such as promoting immunogenic cell death, enhancing antigen presentation, and modulating immune checkpoints. These findings provide a rationale for integrating RT with immunotherapy to improve immune activation and clinical response (39).

Among the most promising strategies is the combination of RT with ICIs, which has shown synergistic potential in overcoming adaptive immune resistance. Parallel efforts to incorporate stroma-targeting agents aim to dismantle physical and biochemical barriers within the TME, thereby enhancing immune cell infiltration and drug delivery. For these approaches to achieve clinical impact, future studies must address critical questions regarding the optimal sequencing of RT and immunotherapy, the development of reliable predictive biomarkers, and the implementation of effective patient stratification frameworks (40).

Moreover, integrating advanced technologies such as single-cell and spatial transcriptomics will be instrumental in identifying actionable immune signatures and guiding personalized combination regimens. Moving forward, a multidisciplinary approach that aligns mechanistic insight with clinical feasibility will be essential to unlock the full therapeutic potential of RT-based immunomodulation in PDAC.
